# It’s Not My Fault: Postdictive Modulation of Intentional Binding by Monetary Gains and Losses

**DOI:** 10.1371/journal.pone.0053421

**Published:** 2012-12-28

**Authors:** Keisuke Takahata, Hidehiko Takahashi, Takaki Maeda, Satoshi Umeda, Tetsuya Suhara, Masaru Mimura, Motoichiro Kato

**Affiliations:** 1 Department of Neuropsychiatry, Keio University School of Medicine, Shinjuku-ku, Tokyo, Japan; 2 Clinical Neuroimaging Team, Molecular Neuroimaging Program, Molecular Imaging Center, National Institute of Radiological Sciences, Inage-ku, Chiba, Japan; 3 Department of Psychiatry, Kyoto University Graduate School of Medicine, Sakyo-ku, Kyoto, Japan; 4 Precursory Research for Embryonic Science and Technology (PRESTO), Japan Science and Technology Agency, Kawaguchi, Saitama, Japan; 5 Department of Psychology, Keio University, Minato-ku, Tokyo, Japan; Ecole Normale Supérieure, France

## Abstract

Sense of agency refers to the feeling that one’s voluntary actions caused external events. Past studies have shown that compression of the subjective temporal interval between actions and external events, called intentional binding, is closely linked to the experience of agency. Current theories postulate that the experience of agency is constructed via predictive and postdictive pathways. One remaining problem is the source of human causality bias; people often make misjudgments on the causality of voluntary actions and external events depending on their rewarding or punishing outcomes. Although human causality bias implies that sense of agency can be modified by post-action information, convincing empirical findings for this issue are lacking. Here, we hypothesized that sense of agency would be modified by affective valences of action outcomes. To examine this issue, we investigated how rewarding and punishing outcomes following voluntary action modulate behavioral measures of agency using intentional binding paradigm and classical conditioning procedures. In the acquisition phase, auditory stimuli were paired with positive, neutral or negative monetary outcomes. Tone-reward associations were evaluated using reaction times and preference ratings. In the experimental session, participants performed a variant of intentional binding task, where participants made timing judgments for onsets of actions and sensory outcomes while playing simple slot games. Our results showed that temporal binding was modified by affective valences of action outcomes. Specifically, intentional binding was attenuated when negative outcome occurred, consistent with self-serving bias. Our study not only provides evidence for postdictive modification of agency, but also proposes a possible mechanism of human causality bias.

## Introduction

The belief that our actions and external events are under control of conscious will is pervasive, and it is rarely doubted [1]. This belief is guided by a feeling that one’s intentional actions caused specific events in the outside world (sense of agency) [2]. Although numerous studies have been carried out to elucidate the cognitive and neural mechanisms of sense of agency, rather less attention has been given to the issue that conscious experiences often provide us with a distorted feeling of causation for voluntary actions and subsequent external events, depending on their rewarding or punishing outcome. One of the most famous examples is self-serving bias; people tend to attribute causes of negative events more to external factors than themselves [3], even though they are indeed responsible for those occurrences, i.e., financial losses in economic activities or an injury to other person following intentional violent acts. In contrast, depressive patients tend to make more realistic assessment of causalities, and are less affected by outcome value [4]. Although such causality bias clearly indicate that representational causal associations between voluntary actions and their consequences are much looser than postulated by current theories, cognitive mechanisms underlying this phenomenon remain unknown.

So far, two potential pathways to the generation of agency have been proposed: prediction and postdiction. According to the former model, a predictive signal of sensory consequence resulting from the action is sent out whenever an action is made. This predictive signal in turn contributes to simulation of the feedback from sensory consequences even before those events occur, and then enhances representational causal linkage between actions and subsequent external events. The predictive model indicates that experience of causation would be constructed at the time of action itself, as an immediate by-product of the physical movement. On the other hand, recent models emphasize retrospective processes that arise after the occurrence of the outcome of action [5], [6]. Accordingly, experiences of causation for voluntary actions and external events are determined in part by post-action information. Such contribution of a retrospective process to conscious experience is often called postdiction, which has been shown to be an important component in the subjective experience of sequential events such as visual motion perception [7] and causality judgment [8].

Several studies have provided supportive evidence for the postdictive explanation. In one study, the experimenters asked participants to report the intentionality of stopping motion during a modified Ouija Board game [9]. When participants were primed with a thought relevant to a subsequent movement, they claimed the movement to have been caused by themselves even though it was actually made by the confederate. This finding suggests that the perceived causal association between intention and subsequent events was determined not by the actual causal relationship, but by the post-action information which was inferred based on the delay between the actual time of intention and the executed action. Another line of evidence for the postdictive account of action awareness comes from the study of choice blindness [10]. In this study, subjects viewed pairs of photographs of females, and were asked to choose which female they found more attractive. Then, the experimenter gave a chosen photograph to the participants, and asked them to give the reasons for having chosen that card. Unbeknownst to the participants, the experimenter swapped cards, and thus presented non-chosen cards to the participants. Although there was a clear mismatch between initial choice and subsequent outcome, participants nevertheless gave introspectively derived reasons for a choice they had not in fact made. This result suggests that people are not intrinsically informed of representational causal association between actions and their consequences, but rather tend to build up inferential accounts based on post-action information. However, the question of how people modify the sense of agency subsequently to rewarding and punishing outcomes of actions remains unsolved.

Importantly, in our daily lives, sense of agency is a pre-reflective and immediate feeling [2], [11], and hence a reliable implicit measure to capture these background experiences is needed. Recent behavioral studies have demonstrated that perceived temporal proximity between action and events plays a pivotal role in the sense of agency [12]. Using a variant of the classical Libet’s clock paradigm of action awareness [13], Haggard et al. reported that voluntary action produces a characteristic distortion of the subjective temporal interval between action and external events [14]. This phenomenon was observed only when the participant’s voluntary actions triggered external events, whereas a muscle twitch in the participant’s finger produced by transcranial magnetic stimulation were less bound to external events. This “intentional binding” phenomenon has been shown to be an implicit measure of agency in a number of studies [15], [16].

The aim of the present study is to investigate postdictive influences of rewarding and punishing outcomes of voluntary action on the sense of agency. Although a single study reported modulation of temporal binding by valences of action outcomes [17], influence of outcome anticipation and valence of outcome were not dissociated. To our knowledge, no one has tested postdictive modification of intentional binding by valence of outcome separately from the influence of anticipation effects. Postdictive account, together with the above-mentioned causality bias, indicates that rewarding or punishing the outcome of action might affect the experience of causation even after the outcome of action has occurred. To test this hypothesis, we conducted a study using a conditioning procedure and a modified intentional binding task combined with a simple gambling game. In this task, voluntary actions yielded positive, neutral or negative monetary payoffs, and thus affective valences of action outcomes were manipulated. Our hypothesis was that temporal linkage between voluntary actions and external events would be modified by affective valences of action outcomes.

## Materials and Methods

### Participants

Twenty-five right-handed healthy subjects participated in the study. They were pre-assessed via a non-structured interview based on DSM-IV TR to exclude current and prior neurological or psychiatric illness. Written informed consent was obtained from each subject. The protocol was approved by the Local Ethics Committee of Keio University School of Medicine, Tokyo, Japan. Three participants were excluded due to highly erratic performance of the modified intentional binding task (standard deviation of mean temporal shifts above 300 ms across trials in one or more conditions) [18]. The final sample therefore consisted of 22 participants (12 females, mean age: *M* = 28.8, s.d. = 4.6 years).

### Tasks and Procedures

#### Acquisition phase

In the acquisition phase, participants underwent conditioning procedures to learn associations between sensory events (tones) and monetary rewards. The procedures of the acquisition phase were based on those of a previous report [19]. Each tone was paired with either a gain of 500 yen, a loss of 500 yen, or no monetary payoff. However, at the beginning, participants were not informed of the associations of tones and monetary payoffs, but rather were instructed to learn them through two sessions of a reaction-time task.

The timeline of the reaction-time task was as follows. Initially, subjects watched a fixation cross on the display. After random intervals (500–1,000 ms), a tone was presented either on the left or right side of headphones. The subject’s task was to respond with a key press as quickly as possible to indicate on which side the tone was presented. Participants were instructed to respond by pressing with their right index finger to indicate ‘left’ or with their right middle finger to indicate ‘right’ within 2,000 ms, and reaction times were recorded. After the participant’s response, visual feedback revealing a monetary outcome paired with the tone was presented for 1,000 ms. The participants performed 30 trials of the reaction time task each in both the first and second sessions. All tones were brief beep sounds of 100-ms duration with a different pitch (300, 1,000 and 3,000 Hz). Pairs of tones and monetary payoffs were counterbalanced across all subjects. The participants were asked to provide a pleasantness rating for each tone from 1 (very unpleasant) to 7 (very pleasant), each in pre- and post-acquisition sessions. Both reaction times and pleasantness ratings were used as indexes of tone-reward conditioning.

#### Modified intentional binding task

After learning tone-reward associations, participants were asked to perform the modified intentional binding task. Initially, an endowment of 3,000 yen was provided to each subject, and they were told that they might lose some or all of this stake, or retain or increase it according to the presented tone. Unbeknownst to the participants, the outcomes of the slots were pre-determined and the total amount of money earned was fixed at 3,000 yen. At the last of the experimental sessions, the participants were debriefed and paid 1,000 yen in compensation.

**Figure 1:**
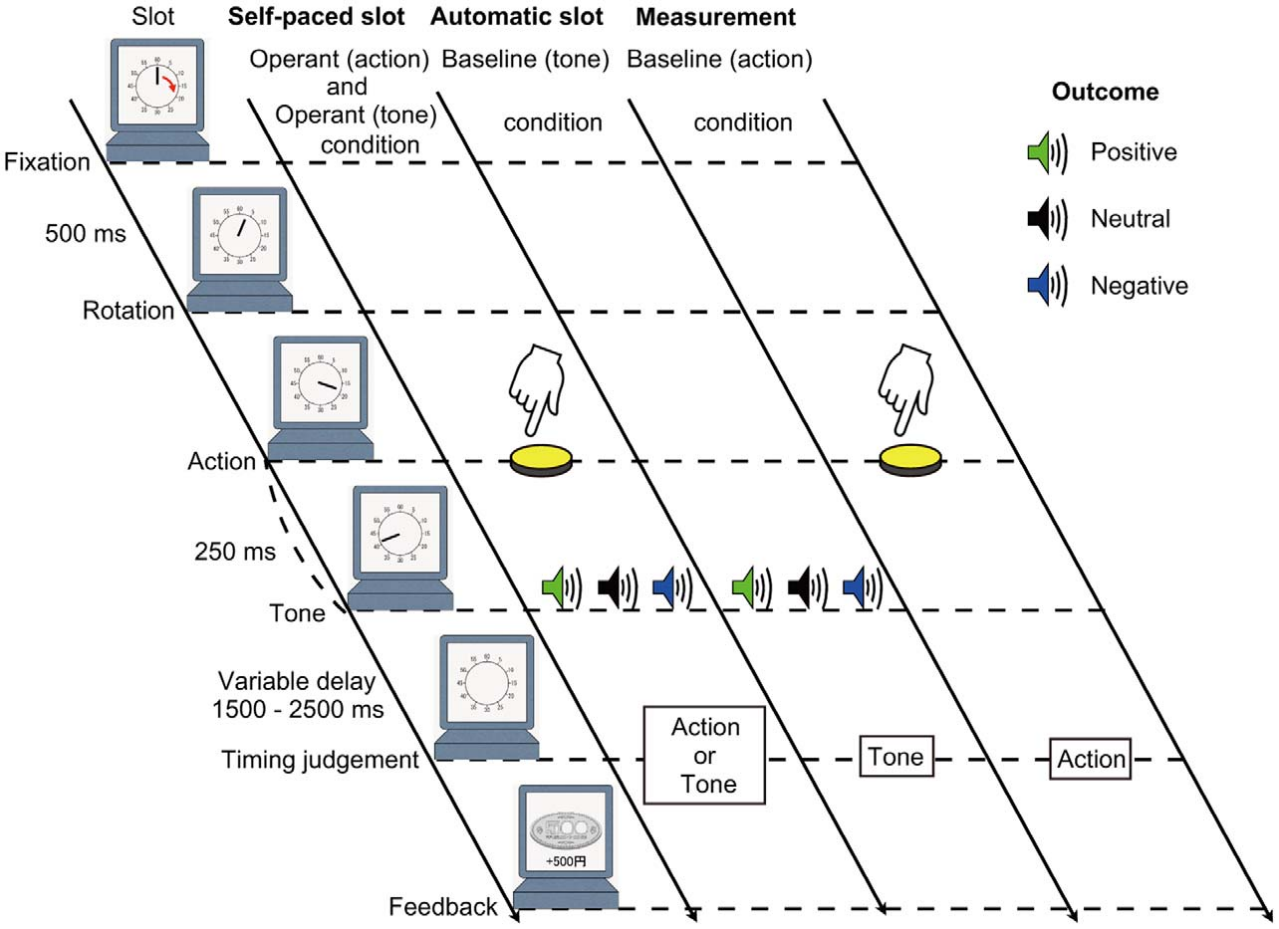
Depiction of the modified intentional binding task. Participants performed the operant (action), operant (tone), baseline (tone) and baseline (action) condition. During each trial, subjects watched a clock-hand rotation and reported the perceived onset time at which a key press or a tone occurred as judged by the perceived position of the clock-hand.

In the present study, the sequence of events from a previous study was employed [14]. Similar to that study, there were four types of conditions: operant (action), operant (tone), baseline (action) and baseline (tone) conditions. However, our experimental design differed in that the participant’s voluntary action caused various monetary outcomes. The sequences of events in the modified intentional binding task are shown in Figure 1. In each condition, a blank screen was first presented for 500 ms, followed by a picture of the slot machine with a clock face and clock-hand. The clock-hand was 12 mm long, which rotated clockwise at a full rotation of 2,560 ms. Initial positions of the clock hand were chosen randomly from 12 conventional 5-min interval positions marked along the circumference of the clock. The clock hand remained stationary at the initial position for 500 ms, and then it began to rotate. Procedures during the clock-hand rotation were as follows. In the operant (action) and operant (tone) conditions, participants performed a voluntary action to yield monetary outcomes. Participants made a key press with the right index finger at a time of their own choosing during the clock-hand rotation. They were instructed to avoid responding at a pre-decided clock position, or during the first half-rotation of the clock hand. Each key press triggered one of three tones after a fixed 250-ms interval. The clock-hand disappeared a random 1,500–2,500 ms after the tone delivery. Participants were asked to report the perceived onset time of their voluntary key press or a tone, as judged by the perceived position of the clock-hand. After this timing judgment, a coin image indicating the amount of monetary payoff was displayed for 500 ms. In the baseline (tone) condition, participants did not press a key but instead waited for a tone to be delivered at a random latency, judging the onset time at which they heard the tone. Therefore, in this trial type, participants were told that monetary outcomes would be determined automatically by the computer at a random time-point. In the baseline (action) condition, participants made a voluntary key press at the time of their own choosing, but it did not yield any monetary outcome. Participants reported perceived onset time of voluntary key press. Each trial type was tested in a separate block, and the order of blocks was counterbalanced across all participants. Participants were instructed to report the onsets of action or tone, at the beginning of each block. Before running the experiment, participants performed 30 practice trials. In the experimental session, the operant (action), operant (tone) and baseline (tone) conditions were tested in 92 trials, which included 32 trials with positive monetary outcome, 30 trials with neutral monetary outcome and 30 trials with negative monetary outcome. In these trial types, the tones were triggered in random order with almost equal probabilities. The baseline (action) condition was tested in 30 trials. All stimuli were displayed using Superlab 4.5 software (Cedrus, Wheaton, MD; Haxby, Parasuraman, Lalonde, & Abboud, 1993).

For data analysis, the perceived time of action or tone in each trial was compared with the actual onset time, and a mean temporal error was calculated for each block [20]. Since our experimental design included different levels of outcomes, temporal binding for each outcome was calculated independently. For example, mean estimate error for actions and tones associated with positive outcome in the baseline condition was subtracted from those associated with positive outcome in the operant condition. Subtracting these baseline estimates allowed us to calculate the shift in the perceived time of the tone and the action associated with positive outcome. These shifts served as measures of action binding and tone-binding for positive outcome, respectively. Finally, the overall binding for positive outcome was calculated as action binding associated with positive outcome minus tone binding associated with positive outcome. Corresponding values for other levels of outcome types were calculated in a similar way. Larger values of this measure indicate stronger intentional binding.

## Results

### Acquisition Phase

#### Reaction times

**Figure 2:**
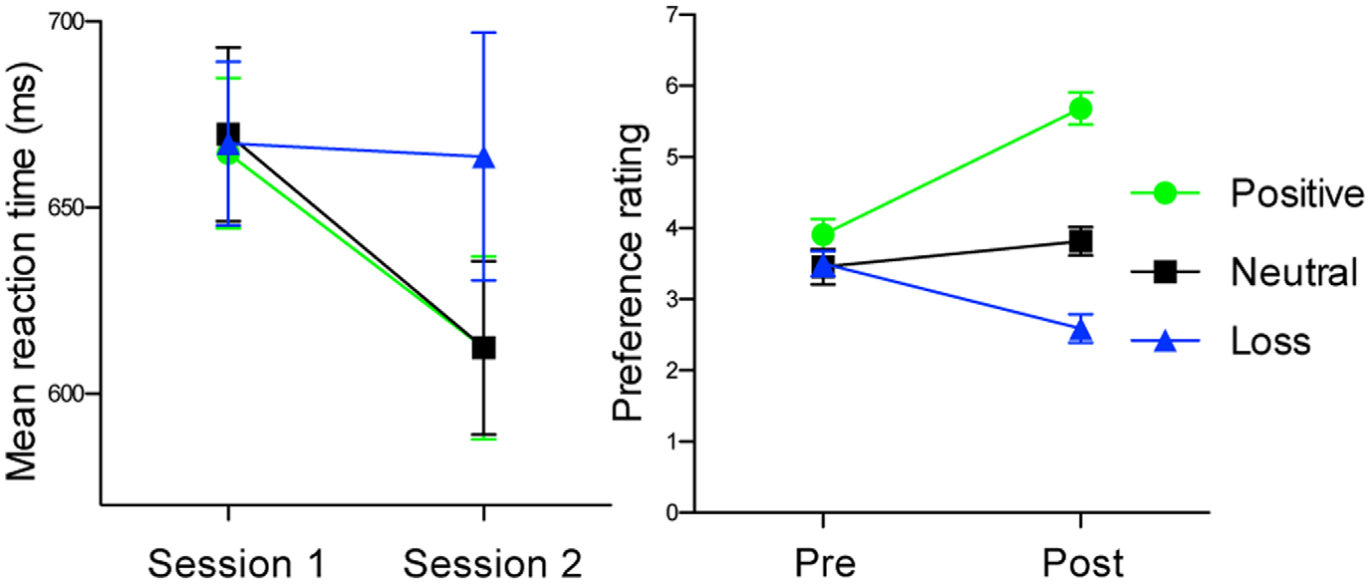
Results in acquisition phase. (**A**) Mean reaction times for auditory stimuli in session 1 and session 2. Bars represent standard errors. (**B**) Pleasantness ratings for auditory stimuli in pre- and post-acquisition sessions. Green, black and blue lines show auditory stimuli paired with positive, neutral and negative outcomes, respectively. Bars represent standard errors.

We examined two behavioral indices of tone-reward conditioning. Initially, we tested differences in reaction times to tones in session 1 and session 2 (Figure. 2a). A 2 (session: session 1 and 2)×3 (valence: positive, neutral or negative) repeated-measures ANOVA of mean reaction times revealed significant valence×session interaction (*F = *4.035, df = 2,42, *p* = 0.025). Multiple comparisons by Bonferroni corrections revealed that responses to negative tones became significantly slower than positive (*t* = 3.375 *p* = 0.001) and neutral tones (*t* = 3.244, *p* = 0.002). These patterns of changes indicate that subjects’ responsivity toward tones changed depending on the monetary outcomes with which auditory stimuli had been paired.

#### Pleasantness ratings

To provide a further behavioral index of conditioning, we tested differences in pleasantness ratings. Results revealed expected patterns of change (Figure. 2b). A 2 (session: session 1 and 2)×3 (valence: positive, neutral or negative) repeated-measures ANOVA of pleasantness rating revealed significant valence×session interaction (*F = *19.796, df = 2,84, *p*<0.0001). Multiple comparisons in post-acquisition session by Bonferroni corrections revealed systematic differences in accordance with valence (*t* = 0.016 to 0.033, *p*<0.0001). These results indicate that pleasantness ratings for the stimuli associated with negative outcome decreased across sessions, while ratings for the stimuli associated with positive outcome increased, demonstrating the development of affective learning over sessions. Taken together, results of the acquisition phase provided evidence that participants changed affective evaluations toward tones as a function of the amounts of monetary outcomes.

### Modified Intentional Binding Task Intentional Binding

#### Overall binding

**Figure 3:**
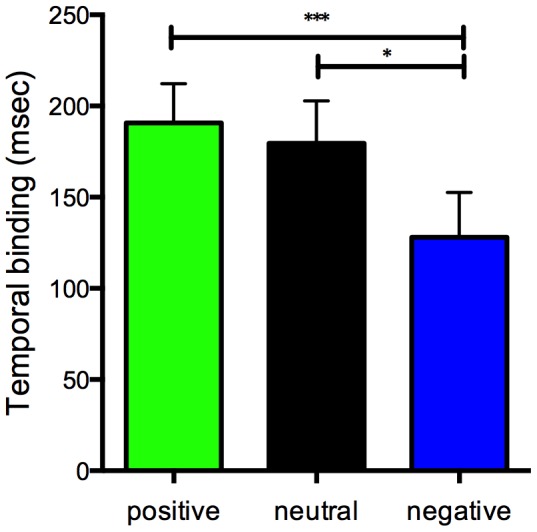
Overall binding for positive, neutral and negative outcomes. Bars represent standard errors.

**Figure 4:**
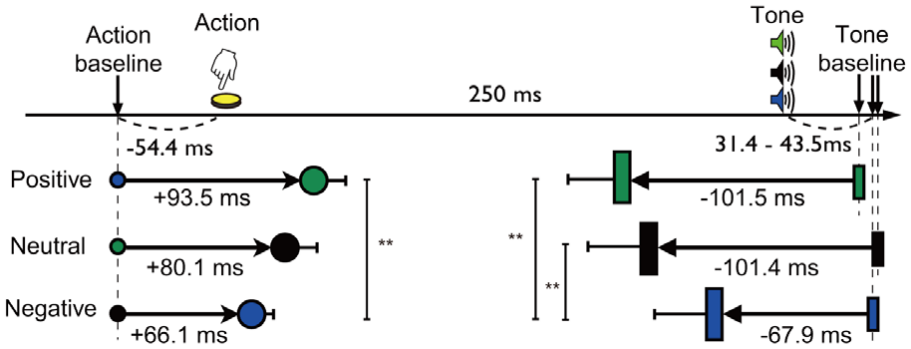
Physical and perceived onset timings of events. From top row, figure shows physical timing of action and tone, perceived timing for positive, neutral and negative outcomes, respectively. Small and large circles show mean perceived timing of action in the baseline (action) condition and the operant (action) condition, respectively. Small and large rectangles show mean perceived timing of tone in the baseline (tone) condition and the operant (tone) condition, respectively. Bars represent standard errors.

We next examined the results of the modified intentional binding task. Intentional binding phenomenon was replicated regardless of the valences of action outcomes (Figure. 3). The main purpose of the present study was to examine the effect of affective valences of action outcomes on intentional binding. A 1×3 (valence: positive, neutral or negative) repeated-measures ANOVA of intentional binding revealed significant main effects by valence (*F = *12.04, df = 2,21, *p*<0.0001), indicating that affective valence of outcome of action influences intentional binding. Multiple comparisons by Bonferroni corrections revealed that overall binding was significantly reduced when negative outcome was triggered as compared to positive (*t* = 3.602, *p*<0.001) and neutral outcome (*t* = 3.602, *p* = 0.003). No significant difference in overall binding was found between positive and neutral outcome (*t* = 0.406, *p* = 0.687). These results indicate that intentional binding was attenuated when voluntary action yielded negative outcomes compared to positive and neutral outcomes. Physical and perceived onset timings of actions and tones in each condition are depicted in Figure 4.

#### Action binding and tone binding

In order to evaluate the influence of the valence of outcome on action binding and tone binding separately, we performed two additional analyses. First, to investigate the influence of the valence of outcome on action binding, we performed repeated-measures ANOVA of action binding with valence (positive, neutral and negative) as within-subject variable. THe results showed a significant main effect of valence (*F = *6.461, df = 2,21, *p* = 0.0036). Multiple comparisons with Bonferroni correction revealed that negative outcome elicited less temporal binding than positive outcome (*t* = 3.594, *p*<0.001). Since there were no differences in the levels of valence in the baseline action condition, differences in action binding could only result from changes in mean estimate errors in the operant condition, demonstrating the valences of outcome modified action binding. The second analysis was performed to investigate the influence of the valence of outcome on tone binding. Repeated-measures ANOVA of tone binding with valence (positive, neutral and negative) as within-subject variable showed a significant main effect of valence (*F = *8.500, df = 2,21, *p* = 0.0008). Multiple comparisons with Bonferroni correction revealed that negative outcome elicited smaller temporal binding than positive (*t* = 3.576, *p*<0.001) and neutral outcome (*t* = 3.567, *p*<0.001). In contrast to action binding, tone binding could arise from changes in mean estimate errors in the baseline or operant conditions. We therefore performed two additional ANOVA analyses to examine judgments errors in the baseline and operant tone conditions separately. A 1×3 (valence: positive, neutral or negative) repeated-measures ANOVA of mean estimate errors for tone in the operant tone condition revealed significant main effects by valence (*F = *6.416, df = 2,21, *p* = 0.0037). In contrast, A 1×3 (valence: positive, neutral or negative) repeated-measures ANOVA of mean estimate error for tone in the baseline tone condition revealed no significant main effect by valence (*F = *0.886, df = 2,21, *p*<0.4200). Thus, the difference in tone binding was mediated by the difference in mean estimate error in the operant tone condition. These analyses indicated that the valence of outcome modulated both action and tone binding.

#### Relation between tone-reward conditioning and intentional binding

The link between tone-reward conditioning and intentional binding was further examined. When change in reaction times and change in preference ratings in the acquisition phase were simultaneously entered as predictors for differences in intentional binding between positive and negative outcomes, a multiple regression equation revealed that prediction of intentional binding by reaction times β = 0.433 (*t* = 1.999, *P* = 0.06), but not preference ratingsβ = 0.055 (*t* = 0.254, *p* = 0.802), was nearly significant. This result implies that modification of action-effect binding is linked to implicit rather than explicit attitude towards sensory outcomes.

#### Precision of timing judgment

It was reported that emotion affects time perception and precision of timing judgment. [21]. To exclude the possibility that difference in intentional binding was due to erratic reports of timing, we assessed standard deviation across trials of timing judgment in each condition. As reported previously, standard deviation of timing judgment provides a general measure of judgment precision [14], [22]. The data are presented in Table 1. For baseline trials, multiple A 1×3 (valence: positive, neutral or negative) repeated-measures ANOVA of standard deviation revealed no significant differences among positive, neutral and negative outcomes (*F = *0.359, df = 2,21, *p* = 0.7003). For operant trials, there were no significant differences in standard deviations among positive, neutral and negative conditions (action: *F = *0.882, df = 2,21, *p* = 0.4216; tone: *F = *1.033, df = 2,21, *p* = 0.3648). These results suggest that precision of timing judgment was not affected by valences of action outcomes. Therefore, it is unlikely that modification of intentional binding is the result of poor attention to spatial memory of the clock hand during the retention interval.

**Table 1:** Mean standard deviation across trials of timing judgment in each condition.

Condition	Event of interest	Outcome	Mean standard deviation across trials
Operant (action)	action	positive	84
		neutral	93
		negative	85
Operant (tone)	tone	positive	84
		neutral	92
		negative	103
Baseline (action)	action	–	79
Baseline (tone)	tone	positive	80
		neutral	74
		negative	81

## Discussion

The main purpose of the present study was to investigate whether sense of agency can be modified in a postdictive manner by affective valences of action outcomes. To examine this issue, we combined intentional binding paradigm with classical conditioning procedures, and thus manipulated monetary outcomes triggered by voluntary actions. Our data showed that intentional binding was attenuated when negative outcomes were caused by subjects’ voluntary actions. The difference in overall binding was mediated by both action binding and tone binding. Note that we varied only the amounts of monetary payoffs triggered by participant’s voluntary key press, and action outcomes were unpredictable at the time of voluntary actions because each monetary payoff was provided in a random order. Therefore, in the present study, action binding was modified mainly by post-action information. Based on previous findings demonstrating intentional binding as a behavioral measure of sense of agency [14], [23], our results support the hypothesis that the sense of agency for self-generated action is built on post-hoc construction at least in part, rather than directly perceived from sensory events. It has been also demonstrated that our conscious experience of causality is not a moment-by-moment construction, but rather is formed by integrating sensorimotor information that occurred within short temporal windows [7], [8]. It should be noted, however, that predictive and postdictive mechanisms are not mutually exclusive. Rather, both predictive and postdictive signals jointly contribute to the generation of agency.

Our data showed that intentional binding was attenuated when negative outcomes were cause by subjects’ voluntary actions compared to positive and neutral outcomes. This result parallels previously reported human causality bias. Past studies have shown that human causality bias is pervasive in the general population [3]. A growing body of evidence has suggested that positivity bias such as self-serving bias is not a mere illusion, but rather has an adaptive feature of human social cognition that is consistently associated with self-protection, maintenance of mental and physical well-being [24]. Other studies reported that self-serving bias is associated with greater self-reported happiness [25], less depression and more positive mood states [26]. In contrast, an absence or attenuation of this bias is indicated to be associated with poor mental health [27]. Thus, a plausible explanation for our results is that modification of the temporal linkage between actions and external events contributes to reduction of the aversive psychological state aroused for unexpected negative events.

In our data, no significant difference in temporal binding between positive and neutral outcomes was found. First, we cannot exclude the possibility that positive stimuli failed to acquire an affective valence that was enough to provide significant behavioral difference due to insufficient repetition of action-outcome parings in the acquisition phase. Indeed, no significant difference in reaction time was observed between positive and neutral tones, which is congruent with the results of intentional binding. However, past studies support that the 60 repetitions of tone-reward parings in the present study are sufficient to produce significant difference in behavioral responsivity [19], [28]. Another possible explanation comes from studies of avoidance learning. Theories of avoidance learning demonstrate that successfully avoiding aversive stimuli becomes rewarding, just as delivery of rewards reinforces operant behaviors [29], [30]. Specifically, it was shown that successful avoidance of monetary losses engages similar neural circuitry as that elicited during monetary gains, thus subserving as intrinsic reward signal [30]. Taking these findings into consideration, it was possible that neutral outcome was perceived as rewarding in the sense of avoiding monetary loss, and therefore neutral outcome elicited similar temporal binding as positive outcome. Although it is difficult to make conclusive remarks on this issue, manipulation of action outcomes with primary rewards such as food or pain relief may solve this problem.

We found larger binding effects compared to those reported in the original intentional binding studies [14], [31]. Earlier studies conducted showed that the magnitude of overall binding ranged from 20 to 61 ms [14], [31]. However, in recent studies with variously modified experimental designs, overall binding was reported to be more than 100 ms [32], [33]. In one study using TMS and shock delivery as effect, overall binding between action and effects was reported to be in a range of 110–150 ms [20]. Another study reported that dopaminergic medication increased the overall binding to be as much as 100–200 ms [22]. Thus, the magnitude of temporal binding using different experimental designs has been highly variable.

Several possible objections should be considered. The first objection to our interpretation could arise from the possibility that reduced intentional binding for negative consequences reflects erratic timing reports due to interference with the retention interval of clock-hand position by arousing stimuli, rather than a specific effect on agency experience. Indeed, several studies have shown that emotion influences temporal attention and time perceptions [21], [34]. If interference effects on time perception modified intentional binding, there would be differences in the precision of temporal judgment. However, our analysis of the standard deviation of timing judgment suggests not; timing judgments were equally consistent among positive, neutral and negative outcomes. Therefore, we provide another explanation, that reward- and punishment-related signals mediate modification of intentional binding. Supporting our interpretation, indirect evidence of modification of intentional binding by predictive reward signal has been presented [35]. Other evidence of modulation intentional binding by reward-related signal was obtained from a study of the effects of dopaminergic agent on intentional binding in patients with Parkinson’s disease (PD). This study showed that dopaminergic medication strengthened intentional binding during the “on” period compared to the “off” period in PD patients [22], indicating that intentional binding is boosted by dopamine. Since dopaminergic signals play a key role in both reward processing and time perception [36], they might mediate modification of intentional binding.

Another possible objection is the link between intentional binding and the sense of agency. As has been pointed out in past reports, the relation between intentional binding and sense of agency is not straightforward [37]. Although it has been reported that intentional binding was closely related to agency in voluntary action [14], [31], [38], several studies have emphasized that causality in more general terms is a primary factor for intentional binding [39], [40]. According to this view, intentional binding should be considered as a special case of a more general phenomenon, causal temporal binding phenomenon. One important difference between agency and causality is that the former presuppose voluntary action or feeling of self-causation, but the latter does not. In one study by Dogge et al., it was shown that intentional binding could occur without voluntary action using modified intentional binding paradigm [41]. In their experiment, participants were asked to make a voluntary key press or engaged an involuntary key press, the latter induced by a magnetic motor system that causes a short depression of the key. In the involuntary movement condition, the feeling of self-causation was manipulated using instructions prior to the intentional binding task. The results showed that strongest binding effects were observed in the voluntary action condition. Furthermore, intentional binding was still observed in the involuntary movement condition. Although this study showed that intentional binding could occur without voluntary action, its magnitude was minimized without feeling of self-causation. In another study using novel temporal binding task, Buehner and Humphreys showed that the causal connection between action and consequence modified temporal binding [39]. Although this study confirmed that causality play an important role in temporal binding, an influence of “self-causation” could not be completely eliminated in that study. The latest study by Buehner attended this issue and provided evidence of temporal binding in the absence of any human action [42]. However, the strongest binding was found when effects were caused by voluntary actions in short action-effect interval. All these results demonstrated that voluntary action is not a sufficient for temporal binding to occur. Nevertheless, these studies also provided constant evidences that voluntary action enhances binding between actions and effects. Although further investigation is needed, the importance of both causality and agency in temporal binding has been highlighted by the study conducted by Cravo et al. [43].

In summary, we investigated how rewarding and punishing outcomes following voluntary action postdictively modulate the sense of agency using intentional binding paradigm and classical conditioning procedures. Our data showed that intentional binding was attenuated when negative outcomes were caused by subjects’ voluntary actions, consistent with self-serving bias. These results provide supportive evidence of a role of postdiction in the experience of agency and a possible source of human causality bias. There are many psychiatric disorders that show an abnormal experience of agency. Several studies have demonstrated enhanced intentional binding in patients with schizophrenia [16], [23] and the prodromal state of psychosis [44]. Such abnormal sense of agency is closely related to core features of schizophrenia. In contrast, depressive patients experience anomaly of agency when their acts caused sensory events that evocated affective responses. They also display depressive realism in their interpretations of events relative to non-depressed individuals that would suggest an attenuation or absence of self-serving bias. We believe that sense of agency would be a key to exploring the psychopathologies of depression and related psychiatric disorders.
